# Erosive tooth wear and use of psychoactive substances among Finnish prisoners

**DOI:** 10.1186/s12903-019-0796-3

**Published:** 2019-05-29

**Authors:** Raija Vainionpää, Kirsi Tuulaniemi, Paula Pesonen, Marja-Liisa Laitala, Vuokko Anttonen

**Affiliations:** 1 Research Unit of Oral Health Sciences, Department of Cariology, Endodontology and Paediatric Dentistry, University of Oulu, P.O. Box 5281, FI-90014 Oulu, Finland; 2 Northern Finland Birth Cohorts, Faculty of Medicine, University of Oulu, Oulu, Finland; 3 Medical Research Centre, Oulu University Hospital and University of Oulu, Oulu, Finland

**Keywords:** Dental caries, Erosive tooth wear, Prison, Prisoners, Psychoactive substances

## Abstract

**Background:**

The aim of the study was to evaluate the prevalence and severity of erosive tooth wear (ETW) and its association with dental caries and the use of psychoactive substances among Finnish prisoners.

**Methods:**

One hundred voluntary prisoners (90.9%) from the Pelso Prison participated in this cross-sectional clinical study between September 2014 and February 2015. Fifty prisoners were also interviewed using the one-on-one interviewing technique for their background factors and use of psychoactive substances. Basic Erosive Index (BEWE) (0–18) was used to measure erosive tooth wear. Decayed (D), missing (M), filled (F) and the number of remaining teeth (T) and DMFT were reported. The association between the different variables was tested and analysed by using cross tabulation. To test the association between the variables a logistic regression analysis was conducted.

**Results:**

Almost all (90%) of the subjects had need for preventive and operative treatment for ETW. In addition, one in five (19%) suffered from severe erosive tooth wear. The use of psychoactive substances and pharmaceuticals is common, yet no association with ETW was found. Smoking and alcohol consumption were more common among younger prisoners than the older ones. There is an increased risk for ETW among older prisoners and major alcohol consumers. Past caries experience was associated with dental erosion.

**Conclusions:**

Erosive tooth wear is common among Finnish prisoners in their thirties. ETW is associated with dental caries and daily alcohol consumption.

## Background

Erosive tooth wear (ETW) includes both chemical and mechanical wear and has a multifactorial and complex aetiology [1]. A recent review article by Schlueter and Luka (2018) proposes the prevalence of ETW to be 20–45% in permanent dentitions [2]. Hyposalivation and frequent and high consumption of acidic drinks and dietary products have been found to be associated with dental caries but also with ETW [3–5]. Specific intrinsic aetiological factors for ETW include eating disorders and problems with acidic reflux disease (gastro-oesophageal reflux disease, GERD) [2, 5]. Alcohol abuse involves both internal (GERD, vomiting) and external factors (alcohol use) and is therefore a risk for ETW [6].

Psychoactive substances include products such as tobacco products, alcohol, illicit drugs and pharmaceuticals, all of which are known to impair oral health [7–9]. Recreational drug use has spread all over the world and the total number of drug users has increased steadily from the 1990s to the 2010s [10]. At the same time, substance abuse, i.e. alcohol consumption and illicit drug use as well as misuse of pharmaceuticals, has increased dramatically among prisoners and is many times higher in comparison with the general population [11]. Prisoners have poorer oral health than the general population [12]. Their oral health-related behaviours are poor per se [12]. They have many other health problems as well, in addition to which alcohol and drug abuse and the use of tobacco products are common [11]. Regular use of medicine is prevalent and there are high levels of psychiatric morbidity among prisoners [13]. However, little is known about ETW as well as the association of ETW and dental caries with psychoactive substance use among Finnish prisoners.

The aim was to evaluate the prevalence and severity of erosive tooth wear (ETW) and the association of ETW and dental caries and the use of psychoactive substances among Finnish prisoners. The hypotheses were that ETW is common among prisoners and an association exists between the use of psychoactive substances and ETW and caries prevalence.

## Methods

### Literature review

Literature on the topic was searched systematically using MeSH keywords in PubMed, MEDLINE and Scopus databases with *prison, prisoner, erosive tooth wear, BEWE* (Basic Erosive Wear Examination)*, dental caries, ICDAS* (International Caries Detection and Assessment System)*, D, M, F, DMFT and psychoactive substance, oral health, illicit drug, dental erosion, alcohol, cannabis, cocaine, methamphetamine, crack* as key words. Only articles in English with adult study populations were included. The time limit was 1999–2018.

### Study population

The participants in this cross-sectional clinical study comprised prisoners in the Pelso Prison, Vaala, Finland. The study population was a comfort sample where all prisoners imprisoned during the period 9/2014–2/2015 were given an opportunity to participate voluntarily. The Pelso Prison is a closed prison which had 110 prison places during the study period. Details of the prisoners’ sentences were not available for the study, but there are more sentenced than remand prisoners in the Pelso Prison.

The invitation for the study yielded a total of 100 out of 110 prisoners. The reasons for not participating were “not having time for this” or “not interested”. Some were transferred to another prison or released, while others just ignored the invitation. The study group was predominantly male (*n* = 89; mean age 35.0 y, min 21 y, max 70 y). The number of females was 11 (mean 37.8 y, min 21 y, max 61 y). Due to the limited number of female prisoners, the data of both genders was combined for analyses. All participants were Finnish citizens. Because of the time limit, out of the 100 clinically examined prisoners, the first 50 (46 men and 4 women, mean age 34.6 y) were invited for interviews on health and health-related behaviours. A one-on-one oral interview with structured questionnaires was used. Interviewing each participant took on average 90 min.

### Dental examinations

All clinical examinations were performed in a fully equipped dental office by the author RV, who was working as a dentist in the Pelso prison during the study. For the oral examination, the light of the dental unit, an oral mirror, a WHO probe and fibre-optic transillumination were used. Individual sterilized (autoclave) instruments were used for all participants. The surfaces of the dental unit and the office were wiped using disinfectant in between patients. These procedures are part of normal protocol in Finland. A three-in-one syringe was used to dry the teeth and teeth were not professionally cleaned before the examination.

### Erosive tooth Wear

The erosive wear of the teeth was measured by using Basic Erosive Wear Examination (BEWE) criteria and was categorized according to the original BEWE scoring system (range 0–3) [14]. All teeth were evaluated for ETW, and the highest score in all the six sextants with at least two teeth (0–3) was determined and the total sum score of the sextants was calculated (range 0–18).

### Dental caries

The International Caries Detection and Assessment System (ICDAS) was used to detect caries lesions [15]. ICDAS is a clinical scoring system that is used to detect and assess dental caries lesions of different stage according to depth and activity. This scoring system can be applied to enamel caries, non-cavitated and manifested lesions. A dental nurse recorded the findings in electronic patient files as called out by the examiner (Effica, Tieto, Oulu, Finland). For recording caries findings per tooth, the ICDAS criteria and activity estimation were used for all dried tooth surfaces. Bitewing radiography was indicated and used when at least one lesion extending into the dentin was observed as recommended by guidelines for controlling dental caries (http://www.kaypahoito.fi/web/kh/suositukset/suositus?id=hoi50078). The radiographs’ information was included in the clinical status. The clinical examination process and the training and calibration protocol have been described in detail elsewhere [12].

### Quality assurance

RV was the examiner and she was trained and calibrated. Before the examinations, an experienced specialist (VA) in similar studies [8] trained the examiner on the study protocol and diagnostic criteria using a PowerPoint presentation. After training, five voluntary randomly selected prisoners were invited to test the protocol and to calibrate and demonstrate the clinical examination. During calibration, RV, VA and MLL carried out the clinical examination. Inter- and intra-examiner agreements (weighted kappa values) were calculated for dental caries and ETW. The kappa values were estimated as follows: > 0.75 excellent reliability, 0.40–0.75 fair to good reliability and < 0.40 poor reliability [16].

To assure the validity of the clinical findings during the six-month study, a total of ten prisoners were examined on three occasions about two months apart by the authors RV (examiner) and MLL (gold standard). For intra-examiner agreement, the author RV re-examined every 10th prisoner approximately one month after the first examination.

### Use of pharmaceuticals

A list of the prisoners’ pharmaceuticals was obtained from the medical records of the participants in Pelso Prison (the prescribed medicines). The pharmaceuticals were categorised as antipsychotics, analgesics, sleeping and falling asleep/insomnia, gastrointestinal, asthma, allergy, and cardiovascular medications, as well as drug-related compensation pharmacy, and others.

### Interview

Because some prisoners were to some extent illiterate, RV interviewed all 50 participants (*n* = 50/100) simultaneously filling in the questionnaire at the dental office. The questionnaire had been validated previously [17]. The clinical examination and the interview were carried out during separate appointments, the clinical examination being done first.

The participants’ age (y*ears)* and gender (*male/female*) were registered. Smoking and the use of snuff, drugs and alcohol in life before imprisonment were surveyed with the following questions: “Do you smoke: *no/1-20 cigarettes daily/more than 20 cigarettes daily*?”, “Have you used snuff in civil life: y*es/no*?”, “Have you used drugs in civil life: *yes/no?”* and *“*Have you used alcohol in civil life: *no/twice a month or less frequently/once a month/every other week/once a week/more than once a week?*” The prisoners were asked the age at which they had started smoking, using alcohol, snuff and drugs as follows: “At what age did you start smoking? ”, “At what age did you start using alcohol?”, “At what age did you start using snuff? ” and “If you use drugs, at what age did you start using them?”(*years of age*).

### Statistics

The prevalence and distribution of the variables were presented as means and standard deviations. BEWE sum scores (0–18) were categorized into three subgroups as suggested earlier [14]: *sum score 0–2* indicating no or only mild erosive tooth wear and no treatment need; *sum scores 3–8* indicating moderate erosive tooth wear and treatment need; and *sum scores 9–18* indicating severe erosive tooth wear and treatment need. The distribution of the highest observed BEWE scores (0–3) for each prisoner was presented.

Caries status was recorded as the frequency for decayed (D), missing (M), restored/filled (F) and remaining teeth (T). Teeth with lesions that had an ICDAS score of active 3 and scores 4–6 were considered needing restorative treatment (D). DMFT values were also calculated. The D values were dichotomised as *< 5 and ≥ 5*, the M values as < *6 and ≥ 6*, the F values as *< 7 and ≥ 7*, the T values as *< 24 and ≥ 24* and the DMFT values as *< 16 and ≥ 16*, according to the distribution of the respective values in the study population.

The association between ETW and psychoactive substances (*alcohol consumption, smoking, and use of snuff and illicit drugs*), pharmaceuticals and dental caries was analysed by using cross tabulation considering the age of the participants with 35 years as a cut-off point. Smokers were divided into non-smokers, smokers (*1–20 cigarettes/day*) and heavy smokers (*more than 20 cigarettes/day*). Subsequently, for the analyses smokers were dichotomized to *yes/no*. As for alcohol consumption, participants were divided into sober *no)*, moderate consumers (*twice a month or less frequently; once a month; every other week; once a week*) and major consumers (*more than once a week).*

The statistical significance of differences between the groups was tested using the Chi-square test or the Fisher’s exact test if the assumptions of the Chi-square test were not fulfilled. To further analyse the association of ETW with other variables, the logistic regression model was carried out with odds ratios (OR) and their 95% confidence intervals (CI). Alcohol, smoking, snuff and drugs were included as explanatory variables in the stepwise logistic regression model. The variables were excluded from the model one by one on the grounds of their significance. In the final logistic regression model, there was alcohol and age. *P*-values < 0.05 were considered statistically significant. All analyses were performed using SPSS (version 24.0; SPSS, Inc., Chicago, Ill., USA).

### Ethics

Participation in the study was voluntary. All participants gave their written informed consent. The Ethical Committee of the Northern Ostrobothnia Hospital District and the Criminal Sanctions Agency gave their approval to the study (respectively, on 16 June 2014 and on 8 August 2014, EETTMK: 50/2014).

## Results

In the literature, only a few articles were discovered concerning prisoners’ psychoactive substance use in association with oral health and only one article investigated erosive tooth wear (ETW) [18] (Table 1). According to the literature, the use of psychoactive substances was common, and the oral health of prisoners was poor in terms of D and DMFT [18–25] (Table 1). The oral health of methamphetamine users was worse than that of other drug users [22, 23] (Table 1). The only article on erosive tooth wear included only cervical lesions and their prevalence was 33%.

**Table 1:** Substance use among prisoners and oral health (DT, DMFT, MT, ETW) (Literature review)

References	Year	Country	Substance use among prisoners	Sample size M male	Age, years Mean (SD)	D/DMF (SD)	Tooth wear/ETW prevalence
Akaji & Folaranmi	2013	Nigeria	52.2% smokers	*n* = 230, M 97%	28 (9.5)	DMF Mean 2 (0.8) Smokers 2 (0.7) Non-smokers 2 (0.8)	–
Almas et al.	2007	Saudi Arabia	98% smokers 61% alcohol drinkers 41% cannabis users	*n* = 100 M 56%	31 (8.9)	–	Cervical erosion/abrasion Total 33% Males 16% Females 55%
Boyer et al.	2015	USA	87% smokers, 58% alcohol consumers, 55% methamphetamine (MA) users, 52% marijuana users, 24% cocaine users, 3% heroin users	MA users *n* = 95 M 79% Non-MA users *n* = 79, M 94%	30 (8.3)	D: MA users 8 (6.2) Non-MA users 6 (5.1)	–
Brown et al.	2013	USA	Alcohol consumers 60%, methamphetamine users 60%	MA users *n* = 59, M 80% Non-MA users *n* = 40, M 80%	33 (10.8) MA users 32 (9.3) Non-MA users 33 (12.8)	D MA users 9 (5.9) Non-MA users 7 (5.4) MA smokers 9 (6.1) Non-oral MA users (intravenous/ intranasal) 7 (5.1) DMF Total 14 (6.7) MA users 15 (6.3) Non-MA users 12 (7.0) MA smokers 16 (6.2) Non-oral MA users (intravenous/ intranasal) 13 (6.3)	–
Heidari et al.	2007	Great Britain	84% alcohol consumers, 78% smokers, 55% cannabis smokers, 66% cocaine, 39% heroin, crack cocaine 12%	*n* = 78	36 (9.6)	D 4 (2.7) DMF 14 (7.5)	–
Osborn et al.	2003	Australia		*n* = 789, M 83%	M 34 (12.4) F 33 (10.5)	D 3 DMF 20	–
Reddy et al.	2012	India	59% beedi tobacco, 7.5% smokers, 6% beedi+smoking, 53% tobacco chewing, 39% tobacco chewing+smoking (beedi = indian tobacco)	*n* = 800, M 90%	41 (12.25)	DMF 5 (2.9)	–
Rouxel et al.	2013	Great Britain	66% current smokers, 11% past smokers, 23% never smoked	*n* = 103, females	31 (9.6)	D 3 (2.5) DMF 12 (7.5)	–

*Beedi:* Indian tobacco product

Among the study population erosive tooth wear was distributed as follows: 19 prisoners (19%) had severe (BEWE sum score ≥ 9), 71% had moderate (BEWE sum scores 3–8) and 10% had mild or no (BEWE sum scores 0–2) erosive tooth wear. Almost all (90%) had the need for preventive and operative treatment (BEWE sum score ≥ 3).

The mean value for the BEWE sum score was 6.6 (SD 2.82). The distribution of the highest BEWE scores of the individuals was as follows: one of the prisoners had score 0, for 50% the highest score was 1, for 46% the highest score was 2, and for 3% it was 3. BEWE scores 2 and 3 were the most prevalent in the anterior sextants both in the mandible (29%) and the maxilla (27%). The mean value for D was 5.1 (SD 5.42) whereas the mean DMFT was 16.8 (SD 8.86) in the entire study population. Four in five prisoners (81%) had the need for the restorative treatment.

Most of the interviewed prisoners (*n* = 50) were smokers (88%) and a fifth (20%) reported using snuff. Out of the heavy smokers, 56% had started smoking under the age of 14. Half of the snuffers had started using snuff at the age of 16 or younger. Almost two-thirds (62%) reported having used illicit drugs at some point of their life and most of the illicit drug users (86%) had started using drugs under 18 years of age. Prior to imprisonment, one in four (24%) had been major consumers of alcohol, and half of them had started drinking alcohol under the age of 15. Almost all (86%) of the younger prisoners (< 35 years) were alcohol consumers. All the younger prisoners (< 35 years) were smokers (*n* = 29) and all the non-smokers (*n* = 6) were older prisoners (≥35 years). On average those consuming alcohol had on average statistically significantly (*p* = 0.002) fewer teeth than those who did not; all those consuming no alcohol had ≥24 teeth (Table 2).

**Table 2:** Prevalence of DT, T, DMFT, age and BEWE and their association with substance abuse among prisoners, n = 50

Variable n (%)		DT n (%)		T n (%)		DMFT n (%)		Age n (%)		BEWE n (%)		
		0–4 27 (54.0)	≥5 23 (46.0)	0–23 17 (34.0)	≥24 33 (66.0)	0–15 20 (40.0)	≥16 30 (60.0)	< 35 29 (58.0)	≥35 21 (42.0)	0–2 2 (4.0)	3–8 37 (74.0)	≥9 11 (22.0)
Alcohol	Major 12 (24.0)	7 (25.9)	5(21.7)	6 (35.3)	6 (18.2)	5 (25.0)	7 (23.3)	7 (24.1)	5 (23.8)	0	7 (18.9)	5 (45.5)
	Moderate 24 (48.0)	13 (48.1)	11 (47.8)	11 (64.7)	13 (39.4)	8 (40.0)	16 (53.3)	18 (62.1)	6 (28.6)	2 (100.0)	19 (51.4)	3 (27.3)
	No 14 (28.0)	7 (25.9)	7 (30.4)	0	14 (42.4)	7 (35.0)	7 (23.3)	4 (13.8)	10 (47.6)	0	11 (29.7)	3 (27.3)
	p	0.913^a^		0.002* ^b^		0.594^a^		0.020 *^a^		0.239^b^		
Smoking	Yes 44 (88.0)	24 (88.9)	20 (87.0)	15 (88.2)	29 (87.9)	19 (95.0)	25 (83.3)	29 (100.0)	15 (71.4)	2 (100.0)	33 (89.2)	9 (81.8)
	No 6 (12.0)	3 (11.1)	3 (13.0)	2 (11.8)	4 (12.1)	1 (5.0)	5 (16.7)	0	6 (28.6)	0	4 (10.8)	2 (18.2)
	p	n.s		n.s		0.381^b^		0.003 * ^b^		0.698^b^		
Snuff	Yes 10 (20.0)	6 (22.2)	4 (17.4)	3 (17.6)	7 (21.2)	4 (20.0)	6 (20.0)	8 (27.6)	2 (9.5)	1 (50.0)	8 (21.6)	1 (9.1)
	No 40 (80.0)	21 (77.8)	19 (82.6)	14 (82.4)	26 (78.8)	16 (80.0)	24 (80.0)	21 (72.4)	19 (90.5)	1 (50.0)	29 (78.4)	10 (90.9)
	p	0.736^b^		n.s		n.s		0.160^b^		0.384^b^		
Drug	Yes 31 (62.0)	15 (55.6)	16 (69.6)	11 (64.7)	20 (60.6)	12 (60.0)	19 (63.3)	21 (72.4)	10 (47.6)	2 (100.0)	25 (67.6)	4 (36.4)
	No 19 (38.0)	12 (44.4)	7 (30.4)	6 (35.3)	13 (39.4)	8 (40.0)	11 (36.7)	8 (27.6)	11 (52.4)	0	12 (32.4)	7 (63.6)
	p	0.309^a^		0.777^a^		0.812^a^		0.075^a^		0.108^b^		

^a^Pearson Chi-Square test, ^b^Fisher’s exact test, *p < 0.05

Major consumer of alcohol: drinking more than once a week; moderate user: once a week/every other week/twice a month or less frequently/once a month

Nearly all (*n* = 87/100) used at least one pharmaceutical and almost one third (*n* = 28/100) had four or more prescribed medications. Half of the prisoners (*n* = 55/100) used antipsychotics, 45% used analgesics, followed by medicines for (sleeping/falling asleep) insomnia (39%), gastro-intestinal problems (27%), asthma (21%), and allergy (10%). Five participants had drug-related compensation medication. Other medication (49%) included skin lotions, medicines for heart disease, cholesterol, hypertension, rheumatoid arthritis and foot ulcers, as well as antibiotics, contraceptives and muscle relaxants.

No association except with alcohol abuse was discovered between the use of psychoactive substances and ETW, nor was the number of prescribed medicines associated with ETW. No association between present restorative treatment need and ETW was found, but the association between filled teeth (F ≥ 7) and erosive tooth wear (BEWE sum score ≥ 9) was statistically significant (*p* = 0.038) (Table 3).

**Table 3:** The association between tooth wear and independent variables, *n* = 100

Variable mean (SD)	n (%)	BEWE n (%)		
		0–8 81 (81.0)	≥9 19 (19.0)	*p*
DT 5.1 (5.42)	< 5 57 (57.0)	47 (58.0)	10 (52.6)	0.669
	≥5 43 (43.0)	34 (42.0)	9 (47.4)	
DMFT 16.8 (8.86)	< 16 46 (46.0)	40 (49.4)	6 (31.6)	0.161
	≥16 54 (54.0)	41 (50.6)	13 (68.4)	
MT 7.7 (6.57)	< 6 49 (49.0)	40 (49.4)	9 (47.4)	0.874
	≥6 51 (51.0)	41 (50.6)	10 (52.6)	
FT 6.9 (5.15)	< 7 53 (53.0)	47 (58.0)	6 (31.6)	0.038*
	≥7 47 (47.0)	34 (42.0)	13 (68.4)	
T 24.3(6.57)	< 23 29 (29.0)	24 (29.6	5 (26.3)	0.774
	≥24 71 (71.0)	57 (70.4)	14 (73.7)	
Age 35.3(10.09)	< 35 53 (53.0)	46 (56.8)	7 (36.8)	0.117
	≥35 47 (47.0)	2 (35 (43.2)	12 (63.8)	

Pearson Chi-Square test, *p < 0.05

The logistic regression analysis revealed an increased risk for ETW among the major alcohol consumers (OR 4.3, 95% CI 0.93–19.44) and older prisoners (> 35 y) (OR 3.5, 95% CI 0.8–15.13).

The intra-examiner agreement was excellent; weighted ĸ - value for BEWE was 0.82 and for ICDAS weighted ĸ = 0.86. The same was true for the inter-examiner kappa values in training; for BEWE weighted ĸ was 0.82 and for ICDAS weighted ĸ = 0.88.

## Discussion

To our knowledge, this is the first paper on dental erosion among prisoners in Finland and in Scandinavia, revealing that ETW is common among Finnish prisoners in their mid-30s. Almost all need either preventive measures and/or operative treatment for erosion. Alcohol seems to be the only psychoactive substance associated with ETW, which is in line with the recent findings of Alaraudanjoki et al. (2016) [26]. Alcohol consumption was also negatively associated with the number of teeth. In addition, those with GI medication had more severe ETW than those without GI- medication.

Lack of international criteria for scoring ETW makes comparing the outcomes of prevalence studies difficult. The commonly used BEWE index and sum score have been shown to be valid for recording ETW, its distribution in dentition and severity [27]. Here, the strength lies in using both BEWE index and sum score. The reliability and reproducibility of the BEWE criteria were supported by excellent intra- and interexaminer agreement.

The prevalence of ETW indicating at least moderate tooth wear among Finnish prisoners was as high as 90%, which is higher than in other recent studies among general populations [26, 28]. Alaraudanjoki et al. (2016) [26] reported 75% prevalence of ETW of some degree among Finns in their mid-40s. In their study, 15% of the individuals with erosive lesions suffered from severe ETW while in the present study the proportion was 19%. In the only study on prisoners and ETW [18] the study population was of similar age, but only cervical erosion/abrasion lesions were recorded (prevalence 33%).

ETW develops over the years but is seen already among children and adolescents as well as in ageing dentitions [29]. Most of the respondents in the present study were 21–35 years of age but were frequently diagnosed with ETW. Presence of ETW among the young is alarming in view of their future oral health. Both Alaraudanjoki et al. [26] and Vered et al. [30] reported that the upper anterior sextants are prone to erosive tooth wear, which is in line with the present study where both the upper and lower anterior sextants were involved.

Erosive tooth wear and past caries experience (F) were statistically significantly associated in this study, whereas Alaraudanjoki et al. (2016) found an association with present restorative treatment need (D) [26]. Lifestyle factors, such as unhealthy dietary habits and acid drinks as well as abuse of alcohol and drugs can be explanatory factors for both dental caries [12] and ETW among this marginal group of people. Moderate and major alcohol consumers had fewer teeth when comparing with the sober participants. Their lifestyle may have favoured only acute dental care; however, this was not studied here.

Over the past 20 years, the prevalence of prisoners’ illegal drug use and drug addiction has increased considerably [31]. Shockingly, two-thirds here reported that they had used illicit drugs at some point of their lives. It has been suggested that parafunctional activity including tooth grinding, clenching and bruxing is associated with psychoactive substances [32] and may worsen ETW. However, no association was found here between ETW and drug abuse.

Almost all participants in the present study population used a prescribed pharmaceutical, antipsychotic medications being the most common group. They are prescribed for recreational drug abuse and personality disorders, which are the two most common diagnoses among Finnish prisoners [33]. Despite the young age of the present study population, one third had more than four prescribed medicines, which is known to cause a risk of hyposalivation and, consequently, dental caries and ETW [34]; however, this association was not found. Saliva is a major protective factor against caries and erosive tooth wear [3]. Not measuring saliva is a limitation of this study and should be included in future studies.

In surveys, the interview is a less frequently used method, but it was valuable among the present study population. Indeed, the interview was the only possibility to gain background information because some of the prisoners had reading and writing difficulties. Interviewing the prisoners took a lot of time but provided more information than could have been obtained from self-written responses. Surprisingly, the prisoners were very open when interviewed about their lives.

Most of the prisoners had started using illicit drugs, alcohol and snuff as well as smoking in their adolescence. The youngest age of smoking initiation was six years. The earlier the substance abuse is started, the more likely it becomes regular [35]; this was true here, too. There is a definite need for preventive means targeted at adolescents and even children regarding psychoactive substances.

In previous studies in prison, the participation rate has been high [36], as is the case in this study as well (91%). The relatively small number of participants, particularly female prisoners, can be considered a limitation. In general, the proportion of women in the prison population is considerably lower than that of men. Another shortcoming is the cross-sectional nature of this study. Therefore, the results concerning substance use and ETW can be regarded as preliminary. In an earlier work on the oral health and oral health-related behaviours of this study population, 6% reported consuming fizzy drinks, whereas consumption of energy or sports drinks was minimal [12]. Therefore, further follow-up studies with larger sample sizes are needed, also including a thorough investigation on other risk factors for ETW in analyses. Interviews were carried out after clinical examinations in a separate appointment due to prison practises; this might have had some influence on the outcome of interviews. Randomized controlled trials (RCTs) with preventive interventions aimed at children and adolescents at risk would be welcome.

## Conclusions

Erosive tooth wear is very common among Finnish prisoners in their thirties; due to the ETW, almost all require both preventive and operative treatment as well as restorative treatment. This study is the first to concern ETW and prisoners in Finland and in Scandinavia. Abuse of psychoactive substances is common among this marginal group and it was therefore investigated as an etiological factor of ETW. The study population here is homogeneous in terms of their health behaviours and educational background [12] and differences within it are difficult to establish, as was reported earlier for dental caries [12] and here for ETW. Prisoners have harmful health behaviours per se*:* they smoke, use alcohol and drugs as well as have poor dietary habits. These indicate a risk for poor health and oral health. Due to their clinical status and health behavioural patterns they also remain at risk for oral diseases in the future.

## Data Availability

In order to protect the participants’ identity, the data will not be made available, but in case of interest for collaboration, please contact the corresponding author.

## Abbreviations

BEWE: Basic Erosive Wear Examination
CI: Confidence interval
DMFT: Decayed, missing, filled teeth
ETW: Erosive tooth wear
ICDAS: International Caries Detection and Assessment System
OR: Odds ratio
SD: Standard deviation

## References

[1] Lussi A, Carvalho TS (2014). Erosive tooth wear: a multifactorial condition of growing concern and increasing knowledge. Monogr Oral Sci.

[2] Schlueter N, Luke B (2018). Erosive tooth wear – a review on global prevalence and its prevalence risk groups. Br Dent J.

[3] Hara AT, Zero DT (2014). The potential of saliva in protecting against dental erosion. Monogr Oral Sci.

[4] El Aidi H, Bronkhorst EM, Huysmans MC, Truin GJ (2011). Multifactorial analysis of factors associated with the incidence and progression of erosive tooth wear. Caries Res.

[5] Schlueter N, Tveit AB (2014). Prevalence of erosive tooth wear in risk groups. Monogr Oral Sci.

[6] Teixeira L, Manso MC, Manate-Monteiro P (2017). Erosive tooth wear status of institutionalized alcoholic patients under rehabilitation therapy in the north of Portugal. Clin Oral Investig.

[7] Jansson L (2008). Association between alcohol consumption and dental health. J Clin Periodontol.

[8] Tanner T, Päkkilä J, Karjalainen K, Kämppi A, Järvelin M-R, Patinen P (2015). Smoking, alcohol use, socioeconomic background and oral health among young Finnish adults. Community Dent Oral Epidemiol.

[9] Rees TD (1992). Oral effects of drug abuse. Crit Rev Oral Bio Med.

[10] World Drug Report 2011. http://www.unodc.org/documents/data-and-analysis/WDR2011/World_Drug_Report_2011_ebook.pdf. Accessed 15 Apr 2019.

[11] Fazel S, Bains P, Doll H (2006). Substance abuse and dependence in prisoners: a systematic review. Addiction..

[12] Vainionpää R, Peltokangas A, Leinonen J, Pesonen P, Laitala M-L, Anttonen V. Oral health and oral-health-related habits of Finnish prisoners. BDJOpen. 2017. 10.1038/bdjopen.2017.6; published online 3 March 2017. 10.1038/bdjopen.2017.6 PMC5842830 29607077

[13] Viitanen P, Vartiainen H, Aarnio J, von Gruenewaldt V, Hakamäki S (2013). Lintonen et al. Finnish female prisoners – heavy consumers of health services. Scand J Public Health.

[14] Bartlett D, Ganss C, Lussi A (2008). Basic erosive and clinical needs. Basic erosive Wear examination (BEWE): a new scoring system for scientific and clinical needs. Clin Oral Invest.

[15] Ismail AI, Sohn W, Tellez M, Amaya A, Sen A, Hasson H (2007). The international caries detection and assessment system (ICDAS): an integrated system for measuring dental caries. Community Dent and Oral Epidemiol.

[16] Fleiss JL, Levin B, Paik MC (2003). Statistical methods for rates and proportions.

[17] Anttonen V, Tanner T, Kämppi A, Päkkilä J, Tjäderhane L, Patinen P (2012). A methodological pilot study on oral health of young, healthy males. Dent Hypotheses.

[18] Almas K, Al Wazzan K, Al Hussain K, Al-Ahdal K, Khan K (2007). Temporomandibular joint status, occlusal attrition, cervical erosion and facial pain among substance abusers. Odontostomatol Trop.

[19] Akaji E, Folaranmi N (2013). Tobacco use and oral health of inmates in a Nigerian prison. Niger J Clin Tract.

[20] Boyer EM, Thompson N, Hill T, Zimmerman M (2015). The relationship between methamphetamine use and dental caries and missing teeth. J Dent Hyg.

[21] Brown RE, De M, Silverstein SJ (2013). Meth mouth severity in response to drug-use patterns and dental access in methamphetamine users. J Calif Dent Assoc.

[22] Heidari E, Dickinson C, Wilson R, Fiske J (2007). Oral health of remand prisoners in HMP Brixton. London Br Dent J.

[23] Osborn M, Butler T, Barnard PD (2003). Oral health status of prison inmates – New South Wales, Australia. Aus Dent J.

[24] Reddy V, Kondareddy CV, Siddana S, Manjunath M (2012). A survey on oral health status and treatment needs of life-imprisoned inmates in central jails of Karnataka, India. Int Dent J.

[25] Rouxel P, Duijster D, Tsakos G, Watt RG (2013). Oral health of female prisoners in HMP Holloway: implications for oral health promotion in UK prisons. Br Dent J.

[26] Alaraudanjoki V, Laitala ML, Tjäderhane L, Pesonen P, Lussi A, Anttonen V (2016). Association of erosive tooth wear and dental caries in northern Finland birth cohort 1966 - an epidemiological cross-sectional study. BMC Oral Health.

[27] Olley RC, Wilson R, Bartlett D, Moazzez R (2014). Validation of the basic erosive Wear examination. Caries Res.

[28] Muller-Bolla M, Courson F, Smail-Faugeron V, Bernardin T, Lupi-Pegurier L (2015). Dental erosion in French adolescents. BMC Oral Health..

[29] Salas MM, Nascimento GG, Huysmans MC (2015). Estimated prevalence of erosive tooth wear in permanent teeth of children and adolescents: an epidemiological systematic review and metagression analysis. J Dent.

[30] Vered Y, Lussi A, Zini A, Gleitman J, Sgan-Cohen HD (2014). Dental erosive wear assessment among adolescents and adults utilizing the basic erosive wear examination (BEWE) scoring system. Clin Oral Invest..

[31] Lintonen T, Obstbaum Y, Aarnio J, Gruenewaldt V, Hakamäki S, Kääriäinen J (2012). The changing picture of substance abuse problems among Finnish prisoners. Soc Psychiatry Psychiatr Epidemiol.

[32] Winocur E, Gavish A, Volfin G, Halachmi M, Gazit E (2001). Oral motor parafunctions among heavy drug addicts and their effects on signs and symptoms of temporomandibular disorders. J Orofac Pain.

[33] Joukamaa M. Health, working capacity and need for treatment of criminal sanction clients. Criminal Sanctions Agency Report 2010, Vammalan Kirjapaino Oy, Vammala, Finland in Finnish http://www.rikosseuraamus.fi/en/index/topical/publications/rise-publications/12010healthworkingcapacityandneedfortreatmentofcriminalsanctionclients.html. Accessed 25 Apr 2019.

[34] Scully C (2003). Drug effects on salivary glands: dry mouth. Oral Dis.

[35] Hawkins JD, Graham JW, Maguin E, Abbott R, Hill KG, Catalano RF (1997). Exploring the effects of age of alcohol use initiation and psychosocial risk factors on subsequent alcohol misuse. J Stud Alcohol.

[36] Andersen HS (2004). Mental health in prison populations: a review- with special emphasis on a study of Danish prisoners on remand. Acta Psychiatr Scand.

